# Studying Biomolecule Localization by Engineering Bacterial Cell Wall Curvature

**DOI:** 10.1371/journal.pone.0084143

**Published:** 2013-12-31

**Authors:** Lars D. Renner, Prahathees Eswaramoorthy, Kumaran S. Ramamurthi, Douglas B. Weibel

**Affiliations:** 1 Department of Biochemistry, University of Wisconsin-Madison, Madison, Wisconsin, United States of America; 2 Technical University Dresden and the Max-Bergmann-Centre for Biomaterials, Dresden, Germany; 3 National Cancer Institute, National Institutes of Health, Bethesda, Maryland, United States of America; 4 Department of Biomedical Engineering, University of Wisconsin-Madison, Madison, Wisconsin, United States of America; University of Texas-Huston Medical School, United States of America

## Abstract

In this article we describe two techniques for exploring the relationship between bacterial cell shape and the intracellular organization of proteins. First, we created microchannels in a layer of agarose to reshape live bacterial cells and predictably control their mean cell wall curvature, and quantified the influence of curvature on the localization and distribution of proteins in vivo. Second, we used agarose microchambers to reshape bacteria whose cell wall had been chemically and enzymatically removed. By combining microstructures with different geometries and fluorescence microscopy, we determined the relationship between bacterial shape and the localization for two different membrane-associated proteins: i) the cell-shape related protein MreB of *Escherichia coli*, which is positioned along the long axis of the rod-shaped cell; and ii) the negative curvature-sensing cell division protein DivIVA of *Bacillus subtilis*, which is positioned primarily at cell division sites. Our studies of intracellular organization in live cells of *E. coli* and *B. subtilis* demonstrate that MreB is largely excluded from areas of high negative curvature, whereas DivIVA localizes preferentially to regions of high negative curvature. These studies highlight a unique approach for studying the relationship between cell shape and intracellular organization in intact, live bacteria.

## Introduction

Bacterial cells control the spatial and temporal organization of their biochemical machinery [1]. The homolog of eukaryotic tubulin, FtsZ and the chemoreceptors are two canonical examples of bacterial proteins that accumulate at the division plane and poles, respectively [2], [3]. Many other bacterial proteins are organized at the subcellular level and a growing number of mechanisms have been hypothesized for controlling their location and function [1], [4]. A key tool in these studies has been the incorporation of fluorescent protein fusions and other optical tags for visualizing proteins using epifluorescence microscopy [5]–[7]. Physicochemical approaches for manipulating cells–particularly those based on microstructured polymers–can complement fluorescent probes and other methods of tracking proteins in cells and provide new opportunities for studying the relationship between cell shape and sub-cellular organization [8]–[10].

Bacteria display a wide range of different cell shapes [11] that are connected to the subcellular localization of cytoplasmic and membrane-associated proteins [12]. Molecular mechanisms that underlie the spatial organization of peripheral membrane proteins in bacteria have been attributed to: i) direct sensing of positive or negative membrane curvature [13]–[15]; and ii) sensing changes in phospholipid (PL) composition in curved membranes [16], [17]. A challenge with the first hypothesis is that individual proteins have length scales that are incompatible with sensing bacterial cell wall curvature, which ranges from ∼0.5–2 µm^−1^. However, the formation of protein complexes and aggregates can create structures with length-scales that are sufficient to sense the mean curvature of bacterial membranes. Several proteins fall into this category and have been reported to have a preference for either positive or negative membrane curvature [13]–[15].

The second hypothesis is based on the introduction of strain in membranes and storing elastic energy in these materials, which alters the local composition of PLs and influences interactions between membranes and proteins [18], [19]. Although this concept is still growing in the biological sciences, it is a widely recognized phenomenon in the branch of materials science and engineering concerned with liquid crystalline materials [20]–[22]. A central feature of the lipid raft hypothesis in eukaryotic cell biology is that changes in the local organization of PLs in biological membranes are correlated with cell shape [23], [24]; a related biophysical phenomenon has been hypothesized to underlie aspects of protein organization in bacteria [25]–[27]. We recently used a microfabrication-based approach to delineate the relationship between membrane curvature and the localization of the anionic phospholipid cardiolipin (CL) in *Escherichia coli* spheroplasts: spherical, osmotically sensitive cells that are formed when the cell wall is removed chemically and enzymatically [28]. Our measurements indicated that the positioning of CL in *E. coli* spheroplasts correlated with negative membrane curvature and was consistent with the reduction of the surface energy potential in strained membranes [16], [29]. A growing number of reported CL-binding proteins in bacteria suggest that this anionic PL may play a central role in organizing biomolecules in response to membrane curvature [28], [30]–[32].

The introduction of methods for controlling cell shape will facilitate the study of these mechanisms in live cells. In this manuscript, we extend two previously reported techniques [28], [33] to explicitly study the relationship between cell shape and the intracellular organization of two proteins that are associated with membranes in regions of bacteria with different cell wall curvatures. First, we grew *E. coli* into filamentous cells in liquid-filled microfluidic channels with user-defined shapes patterned into a layer of agarose. Using these microchannels in combination with epifluorescence microscopy, we found that the cell shape-determining protein MreB fused to red fluorescent protein was distributed along the membrane such that it was preferentially excluded from subcellular regions that corresponded to large values of negative membrane curvature. In unperturbed *E. coli* cells, MreB is typically located along the cylindrical region of bacterial cell walls, whereas the cell division DivIVA of the bacterium *Bacillus subtilis* is positioned at regions of higher negative membrane curvature: primarily at nascent cell division sites and secondarily at the hemispherical cell poles. The largest values of mean curvature imposed on the cell wall using this technique approaches 1 µm^−1^, which is sufficient to mimic cylindrical regions of the cell wall in rod- and crescent-shaped bacteria (∼0.5–1 µm^−1^), but unable to match the curvature of the poles and division septa of bacterial cells (∼2–10 µm^−1^). To transcend this limitation, we used a second technique that enabled us to impose larger values of mean curvature on *E. coli* spheroplast and *B. subtilis* protoplast membranes and observed a correlation between DivIVA localization and negative curvature. Agarose microstructures provide a unique approach for manipulating the curvature of bacterial cell walls and studying connections to biomolecular organization that may elucidate principles of bacterial cell biology.

## Materials and Methods

### Bacterial Strains and Cell Culture

We used the following strains for the experiments described in this paper: *E. coli* strains MG1655 (CGSC 8237), MG1655 pFX40, FB76 [34], DH5α pKR179, and DH5α pKR196, and *B. subtilis* PE103. To construct *B. subtilis* strain PE103, we took advantage of the previously reported construction of *mciZ* under control of the xylose-inducible *P_xyl_* promoter at the *amyE* locus (strain AH93; Handler, Lim, and Losick, 2006). Incorporation of a functional *divIVA-cfp* fusion at the native locus (*divIVAΩlinker-CFP cat*) has been described previously (strain DS4152; Patrick and Kearns, 2008). A list of bacterial strains is summarized in Table 1. We grew bacteria in liquid Luria Bertani broth media (LB; 10 g/L tryptone, 5 g/L yeast extract, 10 g/L NaCl) at 37°C and supplemented the media with 100 µg/mL ampicillin for strains DH5α pKR179 and pKR196, chloramphenicol (30 µg/mL) for strain FB76. LB media containing 1.5% Difco agar (w/v) was used to grow individual colonies of all strains described in this paper. Tryptone, yeast extract, peptone, Petri dishes, and bacteriological agar were from Becton Dickinson (Sparks, MD) and sodium chloride was from Fisher Scientific (Fairlawn, NJ).

**Table 1:** List of bacterial strains used in this study.

Bacterial strain	Genotype	Reference
*E. coli* MG1655	Wildtype K12	lab strain
*E. coli* FB76	*mreB-rfp^SW^ yhdE*<>*cat*	[34]
*E. coli* pKR179	DH5α, *divIVA-gfp*	[13]
*E. coli* pKR196	DH5α, *divIVA-gfp hyperspank*	[13]
*B. subtilis* PE103	*amyE::Pxyl-mciZ cat::spec, divIVAΩlinker-CFP cat*	This study

### Preparation of Bacterial Spheroplast and Protoplasts

We prepared giant spheroplasts of *E. coli* pKR179, pKR196, and MG1655 pFX40 as described previously [28]. In summary, we grew a liquid culture of *E. coli* overnight from a single colony and used a small aliquot (1∶100 dilution) to inoculate liquid LB media. We incubated the culture at 37°C with shaking at 200 rpm and grew cells to an absorbance of 0.5–0.7 (λ = 600 nm). The cell culture was diluted 1∶10 in 4.5 mL of pre-warmed, LB media containing 25 µg/mL cephalexin (Sigma Aldrich, C4895). We incubated the cells for 3–4 h at 37°C with shaking at 200 rpm to grow cells into short filaments. We began monitoring the length of cells after 3 h of growth. When cells reached an average length of ∼50 µm, we harvested them by centrifuging 1 mL of the cell suspension at 3,000×g for 1 min. The pellet was carefully resuspended in 500 µL of a sucrose solution (0.8 M) by gently inverting the test tube several times. We added the following solutions to the aliquot of cells and mixed immediately in between the additions: 30 µL of 1 M Tris-HCl (pH 8.0), 24 µL of 0.5 mg/mL lysozyme (∼20 µg/mL final concentration), 6 µL of 5 mg/mL DNAase (∼50 µg/mL final concentration), and 6 µL of 125 mM EDTA-NaOH (pH 8.0) (∼1.3 mM final concentration). We incubated the mixture for 5–10 min at 25°C and added 100 µL of STOP solution (10 mM Tris-HCl, pH 8, 0.7 M sucrose, 20 mM MgCl_2_) to terminate the digestion. We confirmed the formation of spheroplasts by optical microscopy. Spheroplasts were aliquoted into tubes and used directly.

We formed protoplasts of *B. subtilis* PE103 by modifying the procedure of Schaeffer et al. [35]. A single colony of *B. subtilis* PE103 was inoculated into 2 mL of LB and incubated overnight at 30°C. We used the saturated overnight culture to inoculate LB and grew the culture to an absorbance of 0.5–0.6 (λ = 600 nm). We diluted a small aliquot of the culture 1∶10 into pre-warmed LB medium (total volume, 5 mL) and initiated cell filamentation by adding xylose (20 mM, final concentration). We monitored the growth and filamentation of cells every 30 min for 1–4 h. When cells had reached a length of ∼50 µm, we centrifuged 1 mL of the culture, and resuspended the cell pellet in 1 mL 1xPBS. After this step, we centrifuged the cells, removed the supernatant, and resuspended cells in 200 µL of SM buffer (0.5 M sucrose, 20 mM MgCl_2_, and 10 mM potassium phosphate, pH 6.8). We added lysozyme to the SM buffer to a final concentration of 0.2 mg/mL and incubated the solution for at least 30–60 min at 37°C with shaking at 200 rpm. We monitored the progress of protoplast formation using brightfield microscopy. Lysozyme treatment was continued until the *B. subtilis* filaments had been transformed into protoplasts. We centrifuged protoplasts at 7,000 rpm, discarded the supernatant, and resuspended the pellet in 40 µL of SM buffer.

### Fabrication of Agarose Microchannels

We designed patterns of microchannels and microchambers in CleWin (Delta Mask, The Netherlands) and incorporated them into a chrome mask. Using photolithography, we transferred the pattern from the mask into a 2.7-µm thick layer of Shipley photoresist 1827 that was cast on a silicon wafer; the resulting pattern was embossed into the photoresist. We silanized the resulting photoresist master for 8 h using a vapor of (tridecafluoro-1,1,2,2-tetrahydrooctyl)trichlorosilane (Gelest, Inc., Morrisville, PA). Using soft lithography [36], we transferred the pattern into the silicone elastomer, polydimethylsiloxane (PDMS) (Sylgard 184, Dow Corning) using a ratio of 10∶1 (base to curing agent), and cured the polymer overnight at 60°C. The resulting PDMS layer contained patterns of microchannels or microchambers in bas-relief and was used as a stamp to emboss a layer of agarose or agar [33]. We poured a hot solution of 3% agarose (EM-2120, Omni-pur, EM Biosciences) or agar (Becton Dickinson) containing xylose, IPTG, and antibiotics (as needed) on PDMS stamps oriented with the features facing up, and cooled them to 25°C to gel the agarose. We cut out the layer of agarose embossed with microchannels or microchambers using a scalpel, added a suspension of bacteria (3–5 µL) to the top surface of the agarose, and trapped cells in the microstructures by placing a #1.5 cover slip in contact with the gel. This process has been described in detail previously for microchannels [8], [33] and microchambers [28].

**Figure 1:**
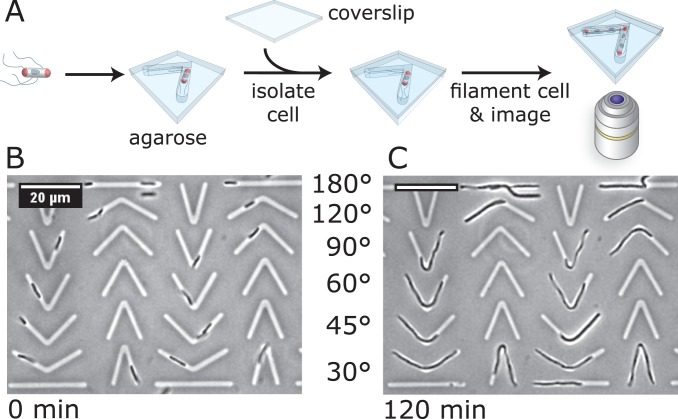
Methodology to engineer curvature in bacterial cells. (A) A schematic diagram depicting the approach for engineering bacterial cell wall curvature by growing filamentous cells of bacteria in angled microchannels. We confined individual planktonic cells in microchannels printed into layers of agarose infused with LB nutrient media to physically impose cell curvature upon filamented bacterial cells. Bacteria were filamented using antibiotics that inhibit cell division. (B–C) Representative images of an experiment using *E. coli* FB76 cells comparing time points at (B) 0 min of growth and (C) 120 min of growth. In this experiment we filamented cells by infusing the agarose with 25 µg/mL of cephalexin. The values shown depict the angle that connects the two straight channel segments. Scale bars: 20 µm.

**Figure 2:**
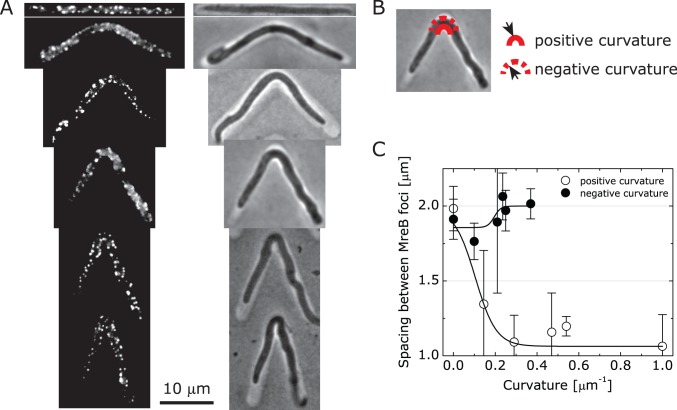
Analysis of MreB distribution in artificially curved bacterial cells. (A) Composite, representative fluorescence and bright field images of filamented *E. coli* FB76 cells in V-shaped channels depicting the spatial distribution of MreB-RFP. Scale bar: 20 µm. (B) An image depicting the positive and negative mean cytoplasmic membrane curvature imposed on the cell. (C) A plot depicting the spacing between MreB foci as a function of mean cell wall curvature determined for cells isolated in V-shaped microchannels and imaged using fluorescence microscopy. The plot depicts the spacing between MreB foci along the positively and negatively curved cytoplasmic membranes in confined cells. The lines between the data points are provided as a guide. For each value of channel curvature, we measured n≥30 cells; error bars represent standard error of the mean.

Using this procedure, we created a pattern of microchannels consisting of six repetitive structures that had a channel length of ∼30 µm, a height of 2.7 µm, a width of 1.6 or 2.5 µm, and a central angle of 30°, 45°, 60°, 90°, 120°, or 180° (Figure 1B). Bacteria growing in these channels became ‘bent’ at the mid-cell and adopted a new region of cell wall curvature corresponding to: 1, 0.54, 0.47, 0.29, 0.145 or 0 µm^−1^, respectively. We determined the mean curvature of the microchambers as shown in Figure S1. Note, that the curvature of the cell wall in the bent region of the channel is not uniform: the ‘inner’ region of the cylinder has a higher mean curvature than the ‘outer’ region (compare Figure 2B). To simplify our analysis, we only characterize the localization of proteins based on the salient curvature of each microchannel. For other studies, however, it may make sense to correlate the position of biomolecules with the inner and outer cell wall curvature. To increase bacterial cell wall curvature beyond the largest values possible using microchannels, we fabricated and used the microchambers described previously (with regions of largest curvature of 1–2 µm^−1^) to confine spheroplasts and protoplasts [28].

### Isolating Cells, Spheroplasts, and Protoplasts in Microstructures

We induced the filamentation of bacterial strains in microchannels by adding cephalexin (25 µg/mL) to *E. coli* or by genetic regulation of division in *B. subtilis.* We grew a single colony of each strain to saturation overnight. The next morning, we diluted the bacterial suspension 1∶100 in fresh medium and grew cells at 37°C and 200 rpm to an absorbance of ∼0.6 (λ = 600 nm). For *E. coli,* we added cephalexin to a final concentration of 25 µg/mL and incubated cells for 5 min at 37°C.

We inoculated a single colony of *B. subtilis* PE103 in 2 mL of LB and incubated at 22°C overnight. The following morning we inoculated 9 mL of LB with 1 mL of the overnight culture (1∶10 dilution). To filament *B. subtilis* cells we transcribed *mciZ*, which codes for a 40-amino acid peptide that inhibits division by preventing the assembly of the FtsZ ring [37]. We grew *B. subtilis* strains to an absorbance of ∼0.6 (λ = 600 nm) and added xylose (final concentration, 20 mM) to induce the transcription of *mciZ* for 5 min before isolating and growing cells in agarose microchannels.

We added 3–5 µL of the bacterial suspension to an agarose pad containing the required antibiotics, IPTG, and/or xylose, and incubated for 1 min. Capillary pressure drew many of the bacteria into the microstructures; some bacteria remained on the agarose surface, which did not complicate the experimental data. We sealed cells, spheroplasts, and protoplasts in microchannels and microchambers by placing a clean #1.5 glass cover slip (12-548-5 g, Fisher Scientific) on top of the layer of agarose.

### Microscopy and Image Analysis

We imaged bacterial cells in microchannels and microchambers on a Nikon TE2000 inverted microscope (Nikon Inc., Melville, NY) equipped with an Andor iXon EMCCD (Andor Technology, South Windsor, CT). Fluorescent probes were excited using a mercury lamp (EXFO Life Sciences, Mississauga, ON, Canada) and the appropriate filters (CFP: λ_ex_ = 433 nm/λ_em_ = 475 nm GFP: λ_ex_ = 484 nm/λ_em_ = 520 nm; RFP: λ_ex_ = 555 nm/λ_em_ = 620 nm). We merged the raw data in fluorescent images (16 bit, gray value images) with phase contrast bright-field images of the micropatterns and analyzed the composite data using ImageJ (NIH, Bethesda, MD). For experiments with cells growing in microchannels, we used an upright Nikon microscope with a 100× oil immersion objective (NA 1.4, resolution: 1 pixel = 105×105 nm) and analyzed the distribution of fluorescent proteins along the length of cells (also compare Figure S3; an average number of pixels per patch is >4–6 pixels.). For the analysis of MreB cluster density in the microchannels, we have included 6 representative images and the methodology of analysis (Figure S3). We analyzed the number of fluorescent clusters along a drawn line (using ImageJ) at the engineered curvature region of the microchannel (as shown exemplarily in Figure S3) and plotted the density vs. the channel length. We then measured the peak-to-peak distance directly in the diagram using Origin (Origin Lab). We analyzed ∼40 cells per curvature region and plotted the average distance vs. the curvature of the different angular microchannels. We imaged proteins in protoplasts and spheroplasts on an inverted Nikon Microscope with a 100× oil immersion objective (NA 1.4, resolution: 1pixel = 165×165 nm). The analysis method we used to determine the spatial localization of proteins has been described previously [28]. Briefly, for experiments with DivIVA in *E. coli* spheroplasts and *B. subtilis* protoplasts, we analyzed the data of the distribution of fluorescent proteins by segmenting spheroplast/protoplasts images and determined the fluorescence intensity along the surface area of each segment. We fit the line scan data to a Gaussian function and determined the position of the fluorescent proteins by calculating the centerpoint of the Gaussian. We performed 10 independent measurements using 10 independent spheroplast or protoplast preparations and analyzed >100 *E. coli* spheroplasts or *B. subtilis* protoplasts for each of the microstructures that we tested. The microstructures were imaged directly through the coverslip.

## Results

### Control of Bacterial Membrane Curvature using Microchannels

We designed and created 30 µm-long microchannels that contained an angle that varied between 30–180° positioned halfway along the channel (Figure 1A). Confining individual bacterial cells and growing them in V-shaped microchannels printed in a layer of agarose enabled us to control the two-dimensional shape of cells, and importantly, the three-dimensional curvature of the cell wall and membranes.

We used two different approaches to grow cells into filaments in channels: i) a genetic approach that controlled FtsZ assembly in *B. subtilis* cells; and ii) a chemical approach in which cephalexin blocked cell division in *E. coli* cells. Figure 1 summarizes the approach for growing cells in angular microchannels. *E. coli* cells grew uniformly in all of the channel curvatures that we studied–cells often grew through the angle before filling the volume available in a straight channel segment (Figure 1). Bacterial cells adopted the shape imposed on them by the agarose microchannels, however we occasionally observed individual cells doubled-over in channels. The resulting cells had a region of mean cell wall curvature that was significantly larger (∼1.2–1.4 µm^−1^) than we were able to create by growing cells through the ‘V’-shape region (in which the maximum channel curvature was ∼1 µm^−1^). Although interesting and potentially useful because the cell wall curvature was larger than we were able to impose using the V-shaped region of the channel, we were unable to control this process and its infrequent occurrence made it an unreliable approach to engineer membrane curvature in our experiments.

As *B. subtilis* cells grew into filaments in V-shaped channels, they filled one of the two straight segments of the channel first before growing through the angled region. We were unable to consistently grow *B. subtilis* cells through channels with angles <120°, which may be due to the thickness of their cell wall (∼30 nm, [38], [39])–and hence their stiffness [40]–compared to *E. coli* cells (∼2–4 nm, [41]).

### MreB Localization is Dependent on Positive Membrane Curvature in *E. coli* Cells with Engineered Curvature

We used the microchannel approach to control membrane curvature and determine how the topology of the cell wall is connected to the positioning of a prokaryotic homolog of eukaryotic actin, MreB. MreB binds to the inner leaflet of the cytoplasmic membrane directly [42], forms small protein complexes in *E. coli* and *B. subtilis* cells that migrate along the inner leaflet of the cytoplasmic membrane [43]–[45], and plays a role in establishing and maintaining the rod shape of bacilli [46]. The motion of the MreB complex is coupled to the assembly of peptidoglycan in the cell wall, and has been described as one of the regulators of cell shape [47]. The broad connections between MreB, peptidoglycan assembly, and cell shape motivated us to study the correlation between its position and cell wall curvature. A constant concentration of MreB on the cell wall may arise from the correlation between MreB and cell wall assembly. We tested this hypothesis by manipulating cell shape, measuring the spatial distribution of MreB along the two opposing cylindrical walls of bacterial cells, and comparing the influence of curvature on the distribution of MreB.

We studied the localization of MreB in *E. coli* FB76 cells isolated and filamented in V-shaped microchannels (Figure 1A). *E. coli* FB76 produces a functional RFP-tagged version of MreB that is expressed under its native promoter, which reduces artifacts associated with overexpression [6]. We observed MreB-RFP foci along the length of *E. coli* cells (Figure 2A). *E. coli* FB76 cells adapted to the shape of the microchannels and formed filaments with a ‘V’-shaped region in the middle of the cell (Figure 1C). Growth through the angled channel produced a region of the membrane with negative curvature and a region with positive curvature. To quantify the localization of MreB and correlate it to cell wall curvature, we analyzed the spatial distribution of MreB foci along the new regions of positive and negative membrane curvature in *E. coli* cells. We chose the spacing between foci as a metric for our measurements because the current model of this protein is that the position of the foci along the cell wall is important for cell wall assembly and provides an indication of how the spacing is influenced by mechanical stress. We observed a consistently smaller number of foci in the negatively curved region of the membrane compared to the positively curved region (Figure 2C). We performed a statistical analysis of the significance of the MreB spacing in different curvature channels (see Figure S2). The spacing between MreB foci in *E. coli* cells confined in microchannels was 1–2 µm at the positively curved region of the cell membrane and ∼1.75–2 µm along the negatively curved region. The spacing of the MreB foci was largely invariant with respect to imposed negative membrane curvature. At larger values of negative membrane curvature, the spacing of MreB foci–which correlates with the concentration of MreB per membrane surface area–increased slightly from 1.75 µm to 2 µm. In contrast, we observed that the spacing between MreB foci decreased from 2 µm to 1 µm (and hence the concentration of MreB associated with membranes) as we increased the magnitude of positive curvature in *E. coli* membranes from 0 to 0.3 µm^−1^. The decrease in the concentration of MreB at increasing values of negative membrane curvature is consistent with the observation that MreB is found primarily along the cylindrical region of the cell wall and is not detected at the hemispherical cell poles, which have a characteristically large mean negative curvature [48]. We analyzed the distribution of MreB foci in the non-curved regions of mechanically manipulated bacteria cells–that is, the spacing of MreB along the cylindrical, non-bent sections in microchannels with angles of 120° to 30°. The average values for the spacing were: 1.99 µm ±0.69 µm (inside) and 2.05 µm ±0.66 µm (outside). These values are approximately identical to the values we measured in straight bacterial cells (compare Fig. 2C).

### DivIVA Distribution in Micromolded Spheroplasts and Protoplasts

To test the influence of cell wall curvature on the influence the localization of a polar protein, we studied the *B. subtilis* division protein DivIVA. DivIVA is a widely conserved membrane-associated protein in Gram-positive bacteria that participates in cell growth and division [49], [50]. Initially, we intended to use the angular microchannel approach to manipulate cell shape and DivIVA-GFP localization. However, we were unable to produce sufficient negative curvature using angular microchannels to influence DivIVA-GFP localization as has been shown in vivo and in silico (Fig. S4) [13], [14] We therefore expressed DivIVA-GFP ectopically in *E. coli* pKR196 and observed the polar localization of DivIVA-CFP fluorescence that is characteristic of the localization of this protein in *B. subtilis* cells. We grew *E. coli* pKR196 cells in the V-shaped microchannels described above and were unable to observe a correlation between DivIVA localization and the imposed curvature created by the microchannels (Figure S4). Presumably, channels with angles ≥30 degrees–corresponding to curvatures of ≤1 µm^−1^–may be too low to influence DivIVA localization [13], [14].

**Figure 3:**
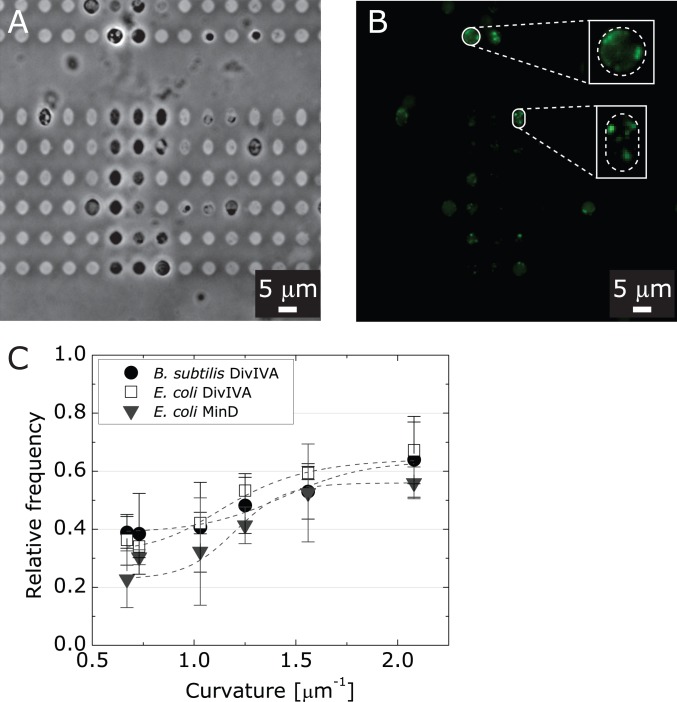
Analysis of DivIVA localization in spheroplasts from filamented cells of *E. coli* pKR196 and protoplasts from filamented cells of *B. subtilis* PE103. (A) A representative brightfield microscopy image of protoplasts from *B. subtilis* PE103 confined in agarose microchambers. (B) A fluorescence microscopy image of DivIVA-CFP in *B. subtilis* PE103 protoplasts confined in agarose microchambers. The images inset show the DivIVA-CFP fluorescence in two magnified protoplasts with imposed membrane curvature that is high and low negative. Scale bars: 5 µm. (C) A plot depicting the relative frequency of the DivIVA distribution in *E. coli* pKR196 spheroplasts (open squares) and *B. subtilis* PE103 protoplasts (shaded circles) and its relationship to microchamber curvature. Data for the relative frequency of MinD-YFP in spheroplasts of *E. coli* MG1655 pFX40 cells (shaded triangles) versus curvature for are provided for comparison to DivIVA-GFP and DivIVA-CFP.

To test whether DivIVA-CFP localizes to artificially curved bacterial membranes, we used a recently reported technique to control the shape of *E. coli* spheroplasts [28] and *B. subtilis* protoplasts to correlate protein fluorescence to imposed membrane curvature. We created spheroplasts and protoplasts from *E. coli* pKR196 and *B. subtilis* PE103 cells from the corresponding cell filaments and confined them in microchambers with a range of shapes/curvatures (Figure 3A). The flexibility of isolated spheroplasts and protoplasts is similar to giant unilamellar vesicles [51] and enables them adopt the imposed shape of the microchambers (Figure 3A). We segmented the confined spheroplasts and protoplasts and analyzed the fluorescence distribution of DivIVA-CFP as described previously [28]. We then analyzed the fluorescence intensity along the long axis of the microchambers (Figure 3B) and plotted the relative frequency of fluorescence versus the curvature along the spheroplast/protoplast membrane (Figure 3C). We defined the curvature as the highest curvature region of the microchambers.

We found that DivIVA-CFP localization in *E. coli* spheroplasts and *B. subtilis* protoplasts depends on the geometry of microchambers and correlates with large values of negative mean curvature: ∼65% of the total DivIVA-CFP fluorescence in spheroplast or protoplasts was localized preferentially to negatively curved regions of membranes (curvature of ∼2 µm^−1^, Figure 3C). The correlation between DivIVA-CFP position and negative membrane curvature was independent of the bacterial strain, as we observed similar patterns of DivIVA-CFP localization in spheroplasts from *E. coli* (strain pKR196) and in protoplasts from *B. subtilis* (strain PE103). Our results confirm earlier reports that DivIVA localizes to geometric constrains in cells that are consistent with a preference for negative membrane curvature [13], [14].

## Discussion

A central hypothesis in cell biology is that cell shape may influence the distribution of biological molecules, including lipids and proteins. We used microchannels to engineer the mean curvature of bacterial cell walls and quantified the spatial distribution of the bacterial proteins MreB and DivIVA. These proteins display different patterns of localization in bacilli: MreB is concentrated along the cylindrical wall and DivIVA is positioned primarily at the division septa and at the cell poles.

MreB is a protein associated with the maintenance of cell shape [52]; deleting MreB or inhibiting its function using small molecules causes rod-shaped cells to become spherical. A long-standing view of MreB was that its assembly into an extended filament formed helical patterns along the cell wall that were important in defining cell shape. Recently, however, MreB was demonstrated to form short polymer fragments that move in trajectories perpendicular to the long axis of the cell [43], [47], [53]. Swulius and Jensen reported that the helical patterns of MreB observed earlier were an experimental artifact of the particular translationally-fused fluorescent protein tag [6]. They found that a different strategy for fluorescently labeling MreB in *E. coli* FB76 cells did not form helices. We previously analyzed the spatial localization of GFP in spheroplasts confined in agarose microchambers and observed a homogeneous distribution of protein with no apparent localization in response to curvature [28]. Due to the overwhelming sequence similarity between GFP and RFP, we expect that the spatial patterns of the two proteins in spheroplasts will be homogenous.

We analyzed the distribution of MreB in *E. coli* FB76 cells using microchannels, microscopy, and image analysis tools to investigate the influence of membrane curvature on this component of the bacterial cytoskeleton. We forced the cell wall of *E. coli* filaments to adopt an externally imposed curvature and found that the pitch between MreB foci is altered compared to the distribution in wild type cells. For the region of the cell wall positioned at the apex of the channel, the spacing between MreB punctae along the positively curved cytoplasmic membrane was dependent on mean curvature while the spacing along the negatively curved membrane was largely independent. Importantly this experiment represents the first demonstration of how the manipulation of the bacterial cell wall curvature in real time can influence the intracellular organization of a membrane-associated protein [46]. Indeed, the preferential exclusion of MreB along more highly negatively curved regions of cells in this experimental system is consistent with the absence of MreB in vivo at the highly concave poles of *E. coli* cells. However, given the current results, we are unable to distinguish whether MreB may directly display a preference for membrane curvature or for particular membrane phospholipids that accumulate in curved membranes. MreB can bind directly to lipid membranes using a N-terminal amphipathic [42], however there is not enough data available to determine the mechanism by which MreB interacts with curved membranes.

DivIVA is a division protein in *B. subtilis*. Ramamurthi et al. and Lenarcic et al. found that DivIVA responds to negative curvature and is primarily localized at actively forming septa of dividing bacterial cells, and secondarily at the hemispherical cell poles [13], [14]. Correct localization of DivIVA is critical for the function of the Min family of division proteins in *B. subtilis* [54]; available in vivo data suggest that the mechanism of DivIVA localization is consistent with sensing membrane curvature. To rigorously test this mechanism in vitro, we manipulated membrane shape by confining *E. coli* spheroplasts and *B. subtilis* protoplasts in microchambers [28]. The magnitude of mean membrane curvature produced using this technique has been <2 µm^−1^–although higher curvatures are possible–and the upper limit is consistent with the mean curvature value of the poles of many rod-shaped bacterial cells. Using this approach for creating larger cell wall curvatures than was possible using microchannels, we confirmed that DivIVA localizes in a manner that is consistent with mean curvature. Increasing microchamber curvature from 0.67 µm^−1^ to 2 µm^−1^ shifted the localization of DivIVA from a random-like distribution (∼30% of the total DivIVA) to a statistically significantly distribution (∼65% of the total DivIVA). Similar results in *E. coli* spheroplasts (MinD localization) and *B. subtilis* protoplasts (DivIVA) confirmed that the curvature-mediated localization of the protein was independent of the bacterial strain. Our in vitro results support the current view of DivIVA localization in bacterial cells and its importance in regulating the division machinery (MinCD) in *B. subtilis* [55].

The localization of biomolecules in bacteria and eukaryotes has been attributed to a number of factors, including geometric cues. Here, we describe a methodology for studying this area of bacterial physiology. Using microchannels to manipulate bacterial shape and study its relationship to protein localization provides a new capability for studying emerging hypotheses in bacterial cell biology. Combined with biophysical methods for probing subcellular organization, we envision this approach will provide opportunities for studying geometrical mechanisms of cell organization–including proteins, phospholipids, and nucleic acids–in a wide range of different microorganisms.

## Supporting Information

Figure S1 **Determination of curvature of the microchambers: the contour length was marked with a 3-point curve tool, the radius was measured by aligning a sphere into the curve, then the radius was converted into curvature.**
(TIF)

Figure S2 **Mean values for spacing between MreB foci and statistical analysis/comparison (t-test) of the spacing of MreB between the positively and negatively curved regions of the bacterial cells.** Comparison between data points with asterisks is statistically significant (***P<0.001, one sample t-test, null hypothesis: mean value negative curvature for each angle value).
(TIF)

Figure S3 **Examples of MreB distribution analyses.** We analyzed the number of fluorescent clusters along a drawn line at the engineered curvature region of the microchannel (as shown exemplarily in Figure S3, the red line representing the outer and the black line representing the inner curvature) using ImageJ (NIH, Bethesda, MD) and plotted the density versus the channel length. We then measured the peak-to-peak distance directly in the diagram using Origin (Origin Lab). We analyzed up to 40 cells per curvature region and plotted the average distance versus the curvature of the different angular microchannels.
(EPS)

Figure S4 **Analysis of DivIVA distribution in angular microchambers of filamented** ***E. coli*** **pKR179 and pKR196.** ***E. coli*** **pKR179 expresses DivIVA from the ectopic locus at basal levels.** The induction levels of DivIVA in *E. coli* pKR196 can be adjusted with different levels of IPTG via a hyperspank promotor. The images are representative bright field and fluorescence images of filamented *E. coli* pKR179 and *E. coli* pKR196 with 1, 10 and 50 µM IPTG in angled microchambers.
(TIF)
