# Supplementary figures and images for: Studying Biomolecule Localization by Engineering Bacterial Cell Wall Curvature
Source: PLoS One. 2013 Dec 31;8(12):e84143. doi: 10.1371/journal.pone.0084143 (PMC3877235; doi:10.1371/journal.pone.0084143)

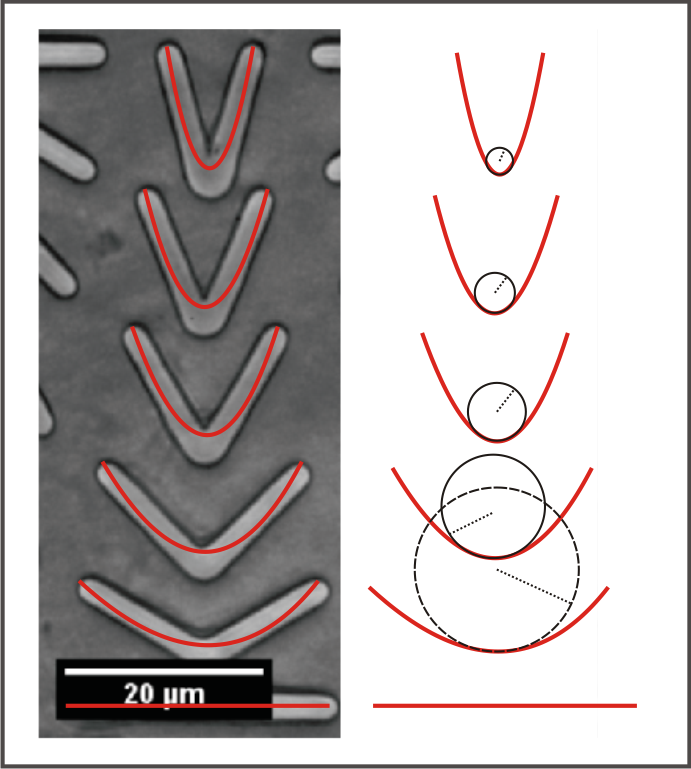

Supplement: Figure S1 — Determination of curvature of the microchambers: the contour length was marked with a 3-point curve tool, the radius was measured by aligning a sphere into the curve, then the radius was converted into curvature. (TIF) [file pone.0084143.s001.tif]

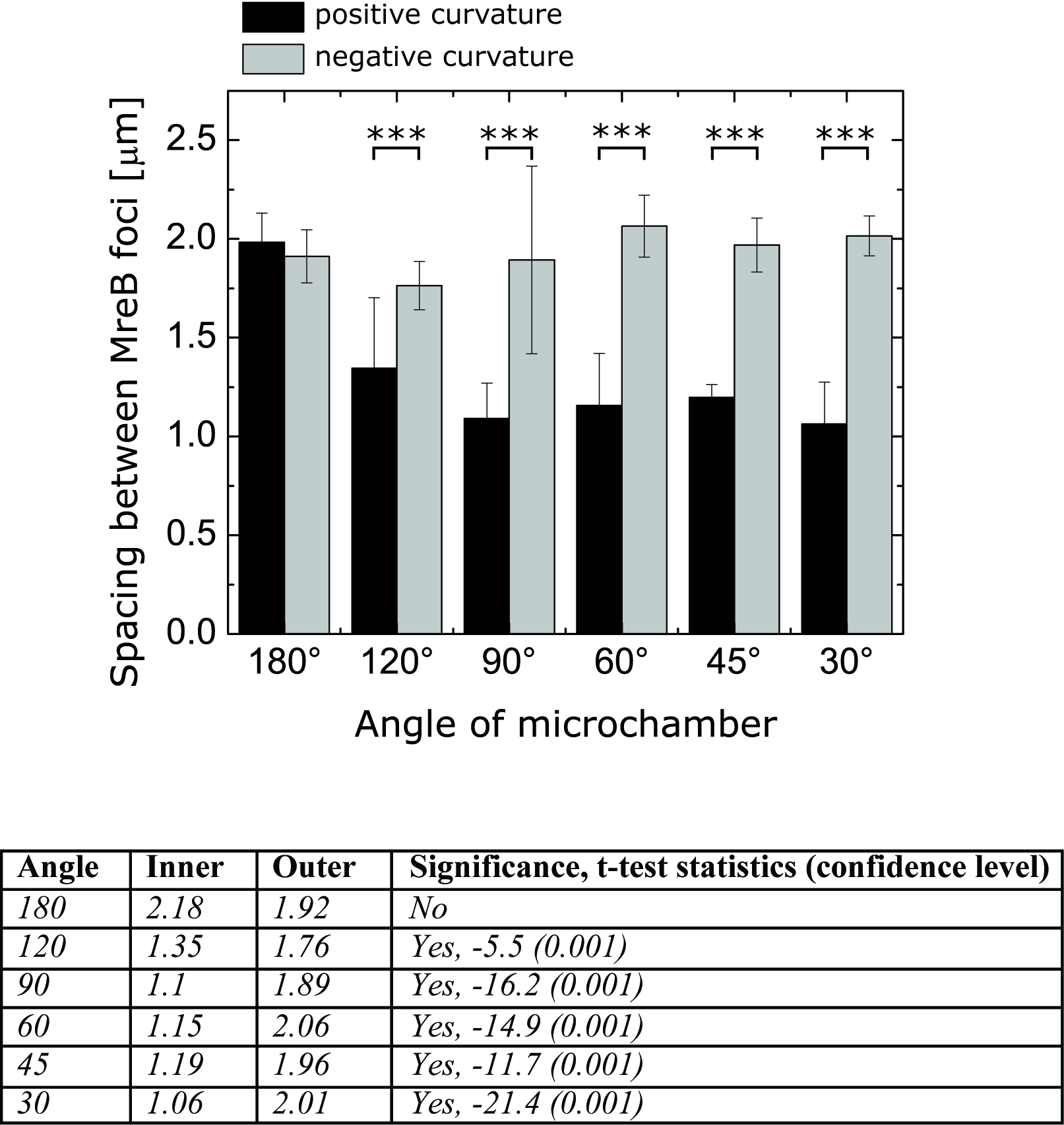

Supplement: Figure S2 — Mean values for spacing between MreB foci and statistical analysis/comparison (t-test) of the spacing of MreB between the positively and negatively curved regions of the bacterial cells. Comparison between data points with asterisks is statistically significant (***P<0.001, one sample t-test, null hypothesis: mean value negative curvature for each angle value). (TIF) [file pone.0084143.s002.tif]

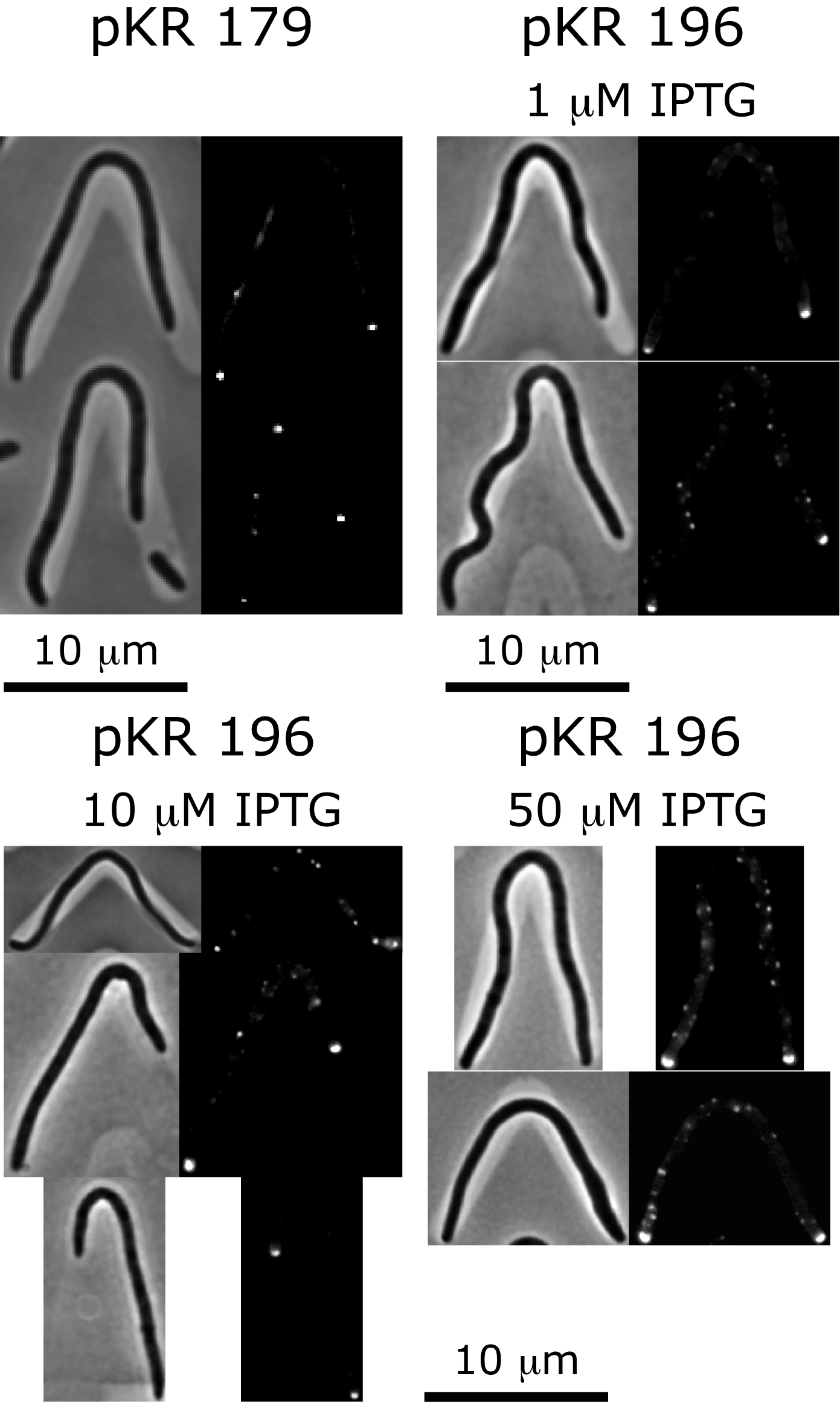

Supplement: Figure S4 — Analysis of DivIVA distribution in angular microchambers of filamented E. coli pKR179 and pKR196. E. coli pKR179 expresses DivIVA from the ectopic locus at basal levels. The induction levels of DivIVA in E. coli pKR196 can be adjusted with different levels of IPTG via a hyperspank promotor. The images are representative bright field and fluorescence images of filamented E. coli pKR179 and E. coli pKR196 with 1, 10 and 50 µM IPTG in angled microchambers. (TIF) [file pone.0084143.s004.tif]
